# Yeast Interactions in Inoculated Wine Fermentation

**DOI:** 10.3389/fmicb.2016.00555

**Published:** 2016-04-22

**Authors:** Maurizio Ciani, Angela Capece, Francesca Comitini, Laura Canonico, Gabriella Siesto, Patrizia Romano

**Affiliations:** ^1^Dipartimento di Scienze della Vita e dell’Ambiente, Università Politecnica delle MarcheAncona, Italy; ^2^Scuola di Scienze Agrarie, Forestali, Alimentari ed Ambientali, Università degli Studi della BasilicataPotenza, Italy

**Keywords:** *Saccharomyces cerevisiae*, non-*Saccharomyces*, yeast–yeast interactions, starter dominance, inoculated wine fermentation

## Abstract

The use of selected starter culture is widely diffused in winemaking. In pure fermentation, the ability of inoculated *Saccharomyces cerevisiae* to suppress the wild microflora is one of the most important feature determining the starter ability to dominate the process. Since the wine is the result of the interaction of several yeast species and strains, many studies are available on the effect of mixed cultures on the final wine quality. In mixed fermentation the interactions between the different yeasts composing the starter culture can led the stability of the final product and the analytical and aromatic profile. In the present review, we will discuss the recent developments regarding yeast interactions in pure and in mixed fermentation, focusing on the influence of interactions on growth and dominance in the process.

## Introduction

During the winemaking process, various microorganisms coexist and interact influencing the dominance, the persistence of fermenting yeasts and the analytical profiles of wine. Although the predominance of *Saccharomyces cerevisiae* on other genera is widely reported (Bisson, 1999; Bauer and Pretorius, 2000), few studies on the competition between species of the same genera (Arroyo-López et al., 2011) and between strains of the same species (Barrajón et al., 2009; Capece et al., 2013; Perrone et al., 2013) are present in literature. On the other hand, as consequence of the re-evaluation of the role of non-*Saccharomyces* yeasts, there is an increasing interest on the use of different species in mixed inoculated fermentation where the yeast interactions play a fundamental role. In this review, we will refer on the recent development regarding the dominance and yeast interactions in inoculated fermentations.

## *S. cerevisiae*/*S. cerevisiae* Interactions

The use of *S. cerevisiae* as starter culture is the most widespread practice in winemaking. However, the inoculation of musts using selected *Saccharomyces* strains does not ensure their dominance at the end of fermentation (Capece et al., 2010). In fact, although possessing high competition, commercial strains do not completely inhibit wild strains until several days after the process has started. The starter culture should compete with not only non-*Saccharomyces* yeasts, but also with indigenous *S. cerevisiae* strains, which theoretically adapt better to must conditions (Barrajón et al., 2011; Capece et al., 2011). The knowledge of the mechanism(s) responsible for interaction among *Saccharomyces* strains could be of particular importance in understanding the observed persistence of these indigenous *S. cerevisiae* strains and the metabolic influence among *S. cerevisiae* strains composing mixed starter cultures. It has been hypothesized that *S. cerevisiae* strains can metabolically interact each other, by modifying fermentation products when grown in mixed culture. A compound, produced by a strain, could be taken up and used by other yeasts present. In this way, yeast interaction and sharing of metabolites could occur. Cheraiti et al. (2005) have demonstrated that redox interactions can occur between yeasts in co-culture, in particular acetaldehyde produced by one yeast was metabolized by the other. This observation provides an explanation as to why modulation of wine flavor in mixed culture cannot be replicated by blending wines together, as the modification arises from complex interactions, largely unknown until now, between strains included in mixed starters (Howell et al., 2006; King et al., 2008; Capece et al., 2013).

**FIGURE 1:**
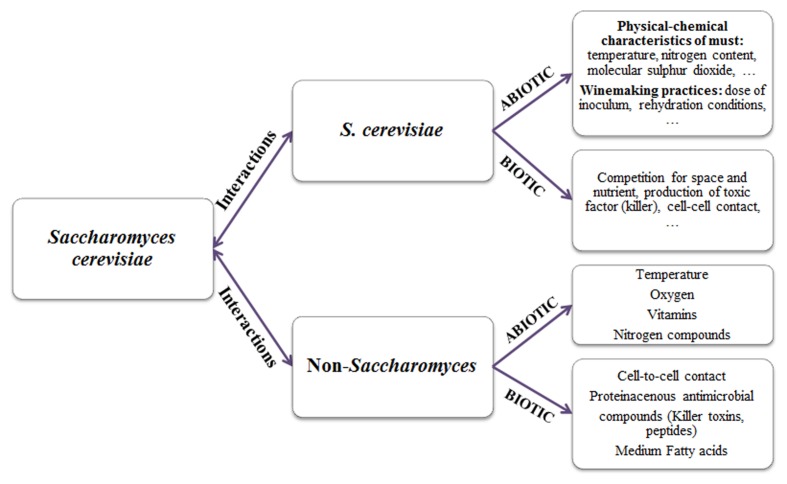
**Factors affecting yeast interactions in inoculated wine fermentation**.

The competition degree of each strain is influenced by a number of abiotic factors (pH, temperature, ethanol, osmotic pressure, nitrogen, molecular sulfur dioxide, etc.) and biotic factors (microorganisms, killer factors, grape variety, etc.), which determine the capacity of one strain to out-compete another (**Figure 1**).

### Abiotic Factors

Some winemaking practices, such as the amount of inoculum, rehydration conditions, or certain physical–chemical characteristics of the must, such as temperature, nutrients (nitrogen, vitamins; Rodriguez-Porrata et al., 2008), led to non-optimal physiological conditions of the starter for competing with the wild biota, causing its growth inhibition by other strains better adapted to a specific oenological environment. Barrajón et al. (2010) evaluated the influence of oenological practices, strain vitality, stress tolerance, and nitrogen requirements on the starters implantation during industrial fermentations. The implantation of commercial strains was generally better in white musts than in red ones, probably in consequence of maceration practice, that might determine an increase of indigenous yeasts in must competing with the starter at the beginning of the fermentation. Different results were obtained: some commercial yeasts competed with one or several dominant wild strains, in other musts the inoculated yeast was completely displaced by only one wild strain at mid-fermentation, for some fermentations a wide variety of wild yeasts was found, none of them dominating the process. Vigentini et al. (2014) have investigated the evolution of the yeast populations during controlled fermentation of Chardonnay musts in two Italian wineries that used the same commercial strain. In the first winery, where the oenologist carefully managed only one starter culture and did not make any spontaneous fermentation, the commercial strain always mastered the process; conversely, in the second winery, where the oenologist performed also spontaneous fermentation, the starter culture did not even take over the dominance and a continuous succession of indigenous strains overcame without one prevailed on the others. Recently, some authors (Duarte et al., 2013) have hypothesized that some oenological additives, such as tannins and fermentation activators, can affect the starter implantation. García-Ríos et al. (2014) carried out a preliminary approach in order to study the fitness advantages of four commercial wine yeast strains (PDM, ARM, RVA, and TTA) against some important oenological parameters, such as nitrogen concentration of grape must, fermentation temperature profile, and ethanol tolerance, which can exert strong stresses on the inoculated strain and determine its competitive advantage. A mathematical approach was used to model the hypothetical time needed for the control strain (PDM) to out-compete the other three strains in a theoretical mixed population. The theoretical values obtained were subsequently verified by competitive mixed fermentations in both synthetic and natural musts, which showed a good fit between the theoretical and experimental data. Specifically, the increase in nitrogen concentration and temperature values improved the fitness advantage of the PDM strain, whereas the presence of ethanol significantly reduced its competitiveness. However, the RVA strain proved to be the most competitive yeast for the three oenological parameters assayed.

Very little is known as fermentation temperature affects the dynamics of the *Saccharomyces* strain population. Torija et al. (2003) studied the influence of fermentation temperature (from 15 to 35°C) on a mixed population of *S. cerevisiae* strains, by evaluating the competition during alcoholic fermentation, at different temperatures, as a tool for testing the natural endurance of indigenous strains. They demonstrated that the temperature of fermentation could clearly affect the development of the different *Saccharomyces* strains: some strains were predominant at low temperatures, whereas others predominated at high ones. The usual growth curve was observed at 25 and 30°C, whereas at 35°C a high yeast mortality was found, which may have induced stuck fermentations with high residual sugar. In fact, these results agree with previous studies, reporting a decrease of yeast viability as the temperature increases (Casey et al., 1984), probably as a consequence of a greater accumulation of intracellular ethanol at higher temperatures, that determine cell toxicity and alter the structure of the membrane, decreasing its functionality (Lucero et al., 2000). On the contrary, at low temperatures there was no decline phase, but the stationary phase lasted until the end of fermentation. The percentage of the different *Saccharomyces* strains changed considerably during fermentation, probably in consequence of their sensitivity to ethanol toxicity. However, it is reported that the ethanol tolerance of some yeast species depends on the temperature (Gao and Fleet, 1988), and this could be the case also for some *Saccharomyces* strains. This may explain why the presence of some strains decreases at higher temperatures, but they are able to finish the fermentation at lower temperatures (Torija et al., 2003).

### Biotic Factors

Ineffective starter implantation was also observed in some fermentation processes despite the use of correct winemaking practices. This means that other factors, i.e., biotic factors, like competition between microorganisms for space and nutrients, or production of toxic compounds (killer factors, medium-chain fatty acids, etc.), can affect starter dominance.

Among the biotic factors underlying the interactions between the different *Saccharomyces* strains during alcoholic fermentation, the killer factor is the most studied. Both neutral and sensitive strains do not produce toxins, but the neutrals are resistant to their action. The use of selected *S. cerevisiae* strains with the killer factor may be effective in suppressing undesirable wild yeast strains or in avoiding stuck fermentations caused by indigenous killer yeasts. The magnitude of killer effect in wine fermentation depends on: the initial ratio of killer to sensitive strains, the presence of protein adsorbing substances, the environmental conditions and the growth phase of the sensitive cells, the presence of protective neutral yeasts, the susceptibility of sensitive strains to the killer toxins of different yeast strains, the inoculum size and nitrogen availability (Pérez et al., 2001). The killer phenotype seems to be linked to the execution of apoptosis, a form of active cell death, widely used by multicellular organisms, e.g., during development or as a mechanism to remove damaged and/or potentially cancerous cells. Apoptotic machinery has been also reported for *S. cerevisiae*. The finding of cell death with apoptosis-like features in yeast (Madeo et al., 1997) was unexpected, as a unicellular organism seems to have no advantages in committing suicide. As the exposure to killer toxins produced and secreted by concurring killer strains is another natural cell death situation for yeast, some authors (Reiter et al., 2005) investigated if killer toxins are able to induce the apoptotic process and if apoptosis is responsible for cell death in the presence of moderate or low toxin concentrations, closely reflecting the situation in the natural yeast habitat. The results showed that killer toxin action can trigger two modes of cell death. Under high toxin concentrations induction of apoptosis plays a minor role, whereas under moderate or low toxin doses, resembling the natural environment of toxin-secreting killer yeasts, induction of apoptosis might play an important role in efficient toxin-mediated cell killing. In this situation, it might be of general importance for a toxin-secreting yeast to induce apoptosis in competing yeast cells, in particular at toxin concentrations that are *per se* too low to kill via the toxin’s primary mode of action.

Another biotic factor involved in the interaction among different yeasts is due to a cell-to-cell contact mechanism. Perrone et al. (2013) investigated *S. cerevisiae* intraspecies competition during wine fermentations, in which the cells of the different strains were mixed or kept separated. In co-fermentation, only the dominant strain was detected, whereas in bio-reactor, in which the cells from the two different strains were kept separate by a membrane and the strains did not sense each other, dominance did not take place. These authors postulated that growth arrest was due to cell-to-cell contact or microenvironment contact; in these conditions, cells compete for space when in high densities and in cell-to-cell contact.

## Non-*Saccharomyces*/*Saccharomyces* Interactions

Controlled multistarter fermentations are characterized by complex interaction between non-*Saccharomyces* and *Saccharomyces* strains (Ciani et al., 2010; Ciani and Comitini, 2015). Although the physiological and biochemical basis for the overall antagonistic interactions among wine yeasts are still unclear, environmental factors, the production of bioactive yeast metabolites or yeast–yeast interaction could be involved (**Figure 1**). In this context, the management of mixed fermentations, such as cell concentration, inoculation modalities (pure or mixed fermentation), and timing of sequential fermentations, require more knowledge on environmental factors and metabolic activities influencing the yeast interactions.

### Management and Yeast Interactions

The management of mixed fermentation strongly influences the dominance and persistence of yeast species. Several investigations showed that in non-*Saccharomyces/S. cerevisiae* co-culture at ratio 1:1, the growth of *S. cerevisiae* was not affected by the co-inoculated yeast, that more or less quickly disappeared. However, at higher inoculation ratio (100:1), *Lachancea thermotolerans* and *Saccharomycodes ludwigii*, *Hanseniaspora uvarum*, and *H. guilliermondii* persisted for more time, while *Candida zemplinina* (synonym *Starmerella bacillaris*) showed a lower competitiveness, increasing its persistence only when the ratio was 10000:1 (Perez-Nevado et al., 2006; Comitini et al., 2011; Domizio et al., 2011). To enhance the competitiveness of non-*Saccharomyces* strains, the sequential fermentation is a useful inoculation modality. The timing of second inoculation, mimicked the spontaneous fermentation, allows to obtain a synergistic interaction between non-*Saccharomyces* and *S. cerevisiae* strains. Several works, investigating on sequential fermentation using various timing of second inoculation, highlighted the actual presence and contribution of several non-*Saccharomyces* species (Andorrà et al., 2012; Azzolini et al., 2012; Gobbi et al., 2013; Canonico et al., 2015).

### Abiotic Factors

As generally recognized, the increasing concentration of ethanol during the fermentation process, is the main factor that determines the dominance of *S. cerevisiae* toward non-*Saccharomyces* yeasts (Pretorius, 2000). Indeed, *S. cerevisiae* strains possess a higher ethanol tolerance than non-*Saccharomyces* yeasts. On the other hand, the competition for nutrients, such as vitamins and nitrogen compounds, contributes to modulate the presence and dominance of yeasts species during wine fermentation (Liu et al., 2015). Oxygen availability affects growth and fermentation performance of wine yeasts having a selective action among the various yeast species (Ciani and Comitini, 2006; Brandam et al., 2013; Jolly et al., 2014; Taillander et al., 2014). Indeed, *S. cerevisiae* and non-*Saccharomyces* wine yeasts exhibit a different behavior in presence of a low oxygen content. In particular, in anaerobic conditions, *S. cerevisiae* is able to grow quickly (Hansen et al., 2001) while non-*Saccharomyces* yeasts belonging to *Hanseniaspora*, *Kloeckera*, and *Torulaspora* genera, grow poorly under the same conditions (Visser et al., 1990). The low competitiveness exhibited by *L. thermotolerans* and *Torulaspora delbrueckii* could be in part explained by their reduced tolerance to scarce oxygen availability (Nissen et al., 2004).

Another important nutrient factor, that could influence the behavior and the dominance of yeast strains in mixed fermentation, is the availability of nitrogen source and vitamins. In general, when non-*Saccharomyces* species grow early during wine fermentation (e.g., spontaneous fermentation), these species can consume amino acids and vitamins, thus limiting *S. cerevisiae* growth (Bisson, 1999; Fleet, 2003). A competition for nutrients was reported by Medina et al. (2012), while Taillander et al. (2014) reported a sluggish fermentation in 48h sequential fermentation of *T. delbrueckii*/*S. cerevisiae* due to nitrogen exhaustion by *T. delbrueckii*. In a recent work, Kemsawasd et al. (2015) indicated that different nitrogen sources had different impacts on the growth and fermentation behavior of *S. cerevisiae* and the other main non-*Saccharomyces* fermenting wine yeasts. On the other hand, non-*Saccharomyces* species and particularly yeast strains belonging to *Hanseniaspora* and *Metschnikowia* genera can contribute to enrichment of the medium as a nitrogen source by their proteolytic activity (Dizzy and Bisson, 2000).

Also the competition for other nutrients may influence the interactions between *S. cerevisiae* and non-*Saccharomyces*. In this context, several positive and negative interactions have been reported regarding substrate limitation or depletion (Ivey et al., 2013; Oro et al., 2014). Among the environmental factors, temperature has an important role in yeast interactions and dominance of the fermentation process. The high temperature in synergy with increasing ethanol concentration affects membrane permeability and integrity. In this contest, some works indicated that ethanol does not provide a clear advantage to *S. cerevisiae* at low temperature (<15°C). Indeed, the persistence and/or the dominance of non-*Saccharomyces* over *S. cerevisiae* at low temperature has been recognized (Gao and Fleet, 1988; Ciani and Comitini, 2006). A study on the interaction between co-inoculated *S. cerevisiae* and *L. thermotolerans* fermentation, showed that the antagonistic effect between these two yeasts were temperature dependent (Gobbi et al., 2013). A recent study, on the evolution of ecological dominance of yeast species, confirmed that temperature of fermentation plays an important role on the ability of *S. cerevisiae* to dominate high-sugar environments (Williams et al., 2015).

### Biotic Factors

The metabolic activities, that influence the controlled multistarter fermentations, could be grouped in antimicrobial molecules and cell-to-cell contact mechanism. Albergaria et al. (2010), investigating on the nature of the toxic compounds produced by *S. cerevisiae* responsible of the early death of *H. guilliermondii* during mixed fermentations, found that the killing effect was due to proteinaceous compounds. In particular, the active proteinaceous compounds exhibited a very low molecular weight that ranged from 2 to 10 kDa and showed a wide antimicrobial spectrum against strains of *Kluyveromyces marxianus*, *L*. *thermotolerans*, and *T. delbrueckii*. Further investigations demonstrated that *S. cerevisiae* during alcoholic fermentation secretes antimicrobial peptides, corresponding to fragments of the glyceraldehyde 3-phosphate dehydrogenase enzyme, that are active against a wide spectrum of wine yeasts including *Dekkera bruxellensis* and the malolactic bacterium *Oenococcus oeni* (Branco et al., 2014, 2015). Among the antimicrobial compounds, killer toxins are certainly involved on the interactions in mixed fermentations. An example of yeast interaction during mixed fermentations non-*Saccharomyces/S. cerevisiae* yeasts due to the action of killer toxin was described by Comitini and Ciani (2010). Another application of non-*Saccharomyces* killer yeasts in sequential fermentation with *S. cerevisiae* starter strain was the use of *Wickerhamomyces anomalus* and *Kluyveromyces wickerhamii* to control *Dekkera/Brettanomyces* spoilage yeasts (Comitini et al., 2004). The main killer toxins involved in wine fermentation are showed in **Table 1**.

**Table 1:** Main killer toxins involved in wine making.

Killer yeast	Killer toxin	Sensitive strain	Applicative indications	Reference
*Saccharomyces cerevisiae* strain “Prise de mousse”	K2 type	*Saccharomyces cerevisiae*	Control of *S. cerevisiae* wild strains	Shimizu, 1993
*Saccharomyces cerevisiae*	K2 type	*Saccharomyces cerevisiae*	Enhance autolysis in Sparkling wine	Todd et al., 2000
*Tetrapisispora phaffii*	Kpkt	*Hanseniaspora*/*Kloeckera*	Control of “apiculate” yeast	Comitini and Ciani, 2010
*Kluyveromyces wickerhamii*	Kwkt	*Dekkera*/*Brettanomyces*	Anti-Brett activity	Comitini et al., 2004
*Wickerhamomyces anomalus*	Pikt	*Dekkera*/*Brettanomyces*	Anti-Brett activity	Comitini et al., 2004
*Pichia membranifaciens*	PMKT2	*Dekkera*/*Brettanomyces*	Anti-Brett activity	Santos et al., 2009
*Torulaspora delbrueckii*	Kbarr-1	*S. cerevisiae* killer strains	Broad anti-wine yeast activity	Ramírez et al., 2015
*Torulaspora delbrueckii*	TdKT	*Pichia* and *Brettanomyces/Dekkera*	Spoilage wine yeasts	Villalba et al., 2016

Together with proteinaceous antimicrobial compounds, medium fatty acids, produced during alcoholic fermentation above a given threshold, could exhibit inhibitory actions toward *S. cerevisiae* and/or other species (Viegas et al., 1989).

Cell-to-cell contact is the other mechanism that could influence the interaction among yeast strains. Nissen et al. (2003) demonstrated this phenomenon carrying out single- and mixed-culture fermentations using both *L. thermotolerans* and *T. delbrueckii* with *S. cerevisiae*.

Similarly, Renault et al. (2013), investigating on the interaction between *S. cerevisiae* and *T. delbrueckii* in a new double-compartment fermenter, found that physical contact between *S. cerevisiae* and *T. delbrueckii* induced a rapid death of the non-*Saccharomyces* yeast. In contrast, when physically separated from *S. cerevisiae*, *T. delbrueckii* maintained its viability and metabolic activity determining a marked impact on *S. cerevisiae* growth and viability. More recently, Kemsawasd et al. (2015) clarified the phenomenon of the early death of *L. thermotolerans* during anaerobic, mixed-culture fermentations with *S. cerevisiae*. They found that this phenomenon was caused by a combination of cell-to-cell contact and antimicrobial peptides.

## Conclusion and Future Perspectives

Investigations on yeast interactions in pure and mixed inoculated fermentation in winemaking are in fast development. Further knowledge on yeast interactions needs to manage the inoculated fermentations, to assure the dominance of inoculated strain in pure fermentation and the contribution of each inoculated yeast in mixed fermentation. In addition, these studies on yeasts interactions will contribute to control undesirable or spoilage microflora avoiding or reducing the use of synthetic antimicrobial compounds, such as sulfur dioxide. As reported above, several features influence the yeast interactions in wine fermentation. To obtain a more complete picture on yeast interaction in inoculated fermentation (pure and mixed with non-*Saccharomyces*) a multifactorial approach using “omics” methodologies should be planned.

## Author Contributions

MC, AC, FC, and PR conceived the idea and outline of the review, LC and GS contributed to the graphical elaboration of data. All authors contributed to writing specific sections and approved the final version of the manuscript.

## Conflict of Interest Statement

The authors declare that the research was conducted in the absence of any commercial or financial relationships that could be construed as a potential conflict of interest.
